# DREAM-*in*-CDM Approach and Identification of a New Generation of Anti-inflammatory Drugs Targeting mPGES-1

**DOI:** 10.1038/s41598-020-67283-0

**Published:** 2020-06-23

**Authors:** Shuo Zhou, Ziyuan Zhou, Kai Ding, Yaxia Yuan, Charles Loftin, Fang Zheng, Chang-Guo Zhan

**Affiliations:** 1 Molecular Modeling and Biopharmaceutical Center, College of Pharmacy, University of Kentucky, 789 South Limestone Street, Lexington, KY 40536 USA; 2 Department of Pharmaceutical Sciences, College of Pharmacy, University of Kentucky, 789 South Limestone Street, Lexington, KY 40536 USA

**Keywords:** Virtual drug screening, Screening

## Abstract

Microsomal prostaglandin E2 synthase-1 (mPGES-1) is known as an ideal target for next generation of anti-inflammatory drugs without the side effects of currently available anti-inflammatory drugs. However, there has been no clinically promising mPGES-1 inhibitor identified through traditional drug discovery and development route. Here we report a new approach, called DREAM-*in*-CDM (Drug Repurposing Effort Applying Integrated Modeling-*in vitro/vivo*-Clinical Data Mining), to identify an FDA-approved drug suitable for use as an effective analgesic targeting mPGES-1. The DREAM-*in*-CDM approach consists of three steps: computational screening of FDA-approved drugs; *in vitro* and/or *in vivo* assays; and clinical data mining. By using the DREAM-*in*-CDM approach, lapatinib has been identified as a promising mPGES-1 inhibitor which may have significant anti-inflammatory effects to relieve various forms of pain and possibly treat various inflammation conditions involved in other inflammation-related diseases such as the lung inflammation caused by the newly identified COVID-19. We anticipate that the DREAM-*in*-CDM approach will be used to repurpose FDA-approved drugs for various new therapeutic indications associated with new targets.

## Introduction

A variety of human diseases, such as the newly identified coronavirus disease 2019 (COVID-19), various forms of pain, cardiovascular diseases, neurodegenerative diseases, and cancers^1–3^, involve serious inflammation conditions without a truly effective and safe anti-inflammatory drug to suppress. Prostaglandin E2 (PGE_2_) serves as the principal proinflammatory agent involved in various inflammation-related diseases^4^. The biosynthesis^5^ of PGE_2_ starts from a polyunsaturated fatty acid, known as arachidonic acid (AA), freed from phospholipids. The AA is first converted to prostaglandin H2 (PGH_2_) by cyclooxygenase (COX)-1 and COX-2^5^. Then, PGH_2_ is converted to PGE_2_ by prostaglandin E synthase (PGES)^6^. The traditional nonsteroidal anti-inflammatory drugs (NSAIDs) either weakly and non-selectively inhibit both COX-1 and COX-2 or potently and selectively inhibit COX-2. All these traditional drugs have a number of serious adverse side effects, including the increased risk of fatal heart attack or stroke and stomach or intestinal bleeding *etc*., because the COX-1/2 inhibition also blocks the synthesis of all of the other prostaglandins (PGs), including PGI_2_, PGD_2_, PGF_2α_, and TXA_2_ that are required physiologically, synthesized from PGH_2_^7^. In comparison with the COX-1/2 inhibition, inhibition of the downstream enzyme, known as microsomal PGES-1 (mPGES-1), will only block the PGE_2_ overproduction without blocking the biosynthesis of the other physiologically required PGs as confirmed by reported knock-out studies^8,9^. So, mPGEA-1, as an inducible enzyme under the inflammation conditions, is a much more promising target for a next generation of anti-inflammatory drugs. One may reasonably anticipate that a highly selective mPGES-1 inhibitor will fully retain the desired anti-inflammatory effects of the COX-1/2 inhibition, but lack of the unwanted adverse effects produced by the inhibition of COX-1/2.

In fact, various inhibitors of human mPGES-1 have been reported in the literature for development of the next-generation inflammatory drugs^10–31^. Unfortunately, it is still rare to see that a potent inhibitor of human mPGES-1 can also potently inhibit mouse or rat mPGES-1. This problem prevents using well-established mouse/rat models of inflammation, pain, and other diseases for preclinical drug development studies^32^. It would be extremely challenging for the mPGES-1-based drug development to follow the traditional drug discovery and development route (which starts from lead identification and optimization based on various *in vitro* and *in vivo* assays in terms of the activity and toxicity *etc*.). Here we report a new approach, called DREAM-*in*-CDM (Drug Repurposing Effort Applying Integrated Modeling-*in vitro/vivo*-Clinical Data Mining), to identify an FDA-approved drug suitable for use as an effective analgesic targeting mPGES-1. The DREAM-*in*-CDM approach consists of three steps: (1) computational modeling to predict which FDA-approved drugs may favorably bind with the desirable drug target (mPGES-1 in this project); (2) *in vitro* and/or *in vivo* assays to validate the computational predictions; (3) clinical data mining to confirm the efficacy associated with the required clinical end points for the new therapeutic indication. By using the DREAM-*in*-CDM approach, multiple FDA-approved drugs (that can be used orally), including lapatinib, have been identified as truly promising mPGES-1 inhibitors that may be repurposed to treat various inflammation-related diseases.

## Results

### Predictions from computational modeling

**Figure 1:**
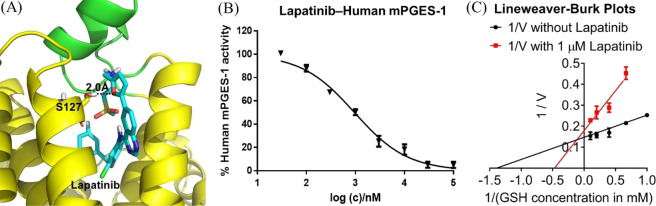
(**A**) Modelled binding structure of lapatinib (cyan sticks) with mPGES-1 (yellow cartoon) obtained by using the crystal structure (4AL0). The cap domain is colored in green and some key residues interacting with lapatinib are showed in sticks. Hydrogen bond is showed in black dashed line. (**B**) Dose-dependent inhibition of lapatinib against mPGES-1. (**C**) Inhibition of mPGES-1 with lapatinib at constant 1 µM concentration and GSH at different concentrations, showing that lapatinib is a GSH-competitive inhibitor.

On the basis of our previous computational modeling studies^32–34^, we recently obtained the open conformation of human mPGES-1 through fully relaxed molecular dynamics simulations^35^. The modeled open conformation of human mPGES-1 was used further to virtually screen FDA-approved drugs for their potential binding with mPGES-1 in this study. The virtual screening was performed by using a multiple-step computational screening protocol (see the Materials and Methods section below). Based on the virtual screening (including molecular docking, energy minimization, MM-PBSA binding free energy calculation, and manual checking of the binding poses for the top-50 compounds), 15 FDA-approved drugs (that can be used orally), including lapatinib, were predicted to be inhibitors of mPGES-1. For further computational testing, we also tested the binding of the 15 drugs with the high-resolution crystal structure (4AL0 with a high resolution at 1.2 Å) of mPGES-1 in which co-factor glutathione (GSH) was removed from the binding site before molecular docking, confirming that these drugs can bind to the GSH-binding site of the crystal structure (see Fig. 1A for the binding mode of lapatinib). So, both the computationally modeled structure and the X-ray crystal structure of mPGES-1 can reasonably accommodate these drugs in the same GSH-binding site.

In addition, we also examined whether lapatinib can fit the active site cavity of COX-2 by performing molecular docking in comparison with celecoxib (a well-known potent inhibitor of COX-2 in clinical use). It turned out that the binding pocket of celecoxib was too tight for lapatinib to fit into. Based on the molecular docking, lapatinib was not expected to be an effective inhibitor of COX-2.

### *In vitro* activity

The above computational predictions were followed by *in vitro* activity assays for their actual inhibitory activity against human mPGES-1 using an ELISA assay described in our previous reports^32–34^. All compounds for the FDA-approved drugs from the Enzo Compound Library were provided with a purity of 95% or better^36^. Specifically, 15 FDA-approved drugs (see Fig. S1 of the Supporting Information for the molecular structures) were assayed for their inhibitory activity against human mPGES-1. According to the initial single-concentration (at 10 µM) screening, the 15 drugs inhibited the mPGES-1 activity by 4% to 99%, and 7 out of the 15 drugs at 10 µM inhibited the mPGES-1 activity by 44% or more. The top-7 drugs were assayed further for their IC_50_ values (see Table 1), with lapatinib being the most potent one (IC_50_ = 0.8 µM or 800 nM). In addition, some other FDA-approved drugs can also significantly inhibit human mPGES-1, but with relatively higher IC_50_ values (lower potency). So, we first focused on lapatinib in further experimental tests in this investigation.

**Table 1:** The FDA-approved drugs selected from virtual screening and tested for their *in vitro* inhibitory activity against human mPGES-1.

Compound Name	Inhibition (%) at 10 µM	IC_50_ (µM)
Lapatinib	99	0.8 ± 0.1
Acitretin	83	3.5 ± 1.6
Calcipotriene	68	3.0 ± 1.1
Trandolapril	54	9.0 ± 2.3
Tolvaptan	52	10.2 ± 2.5
Cefpodoxime	47	29.7 ± 6.3
Prednisolone	44	12.6 ± 1.6
Dipyridamole	36	
Penicillin V Potassium	36	
Estramustine Phosphate	36	
Alfuzosin	22	
Doxazosin	21	
Adefovir Dipivoxil	19	
Alprostadil	11	
Dinoprostone	4	

Further, we determined *in vitro* inhibitory activity of lapatinib against mouse mPGES-1 and obtained that IC_50_ = 12 µM. So, lapatinib has a 15-fold lower inhibitory activity against mouse mPGES-1 compared to human mPGES-1 (IC_50_ = 0.8 µM).

In addition, lapatinib was assayed for its potential inhibitory activity against mixed COX-1 and COX-2 (denoted as COX-1/2) with equal amounts of COX-1 and COX-2 in terms of the enzyme activities. Notably, lapatinib at a concentration of 100 µM did not significantly inhibit COX-1/2, which confirmed the above computational prediction.

### Binding mode analysis

Depicted in Fig. 1 are the computationally modeled protein-lapatinib binding structure (Fig. 1A, showing a favorable hydrogen bond between oxygen on the furan ring of lapatinib and the hydroxyl group of S127 side chain) obtained by using the crystal structure (4AL0) and *in vitro* activity data (Fig. 1B) obtained for lapatinib. To examine whether lapatinib competes with GSH in binding mPGES-1, we tested the mPGES-1 enzyme activity (enzymatic reaction velocity V) with various GSH concentrations in the presence and absence of 1 µM lapatinib to determine the Lineweaver-Burk plots (Fig. 1C). As well-known, by using a Lineweaver-Burk plot, one can obtain the Michaelis-Menten constant (*K*_M_) from the *x*-intercept (*i.e*. the *x*-intercept = −1/*K*_M_) and V_max_ from the *y*-intercept (*i.e*. the *y*-intercept = 1/V_max_). Thus, we obtained *K*_M_ = 2.1 mM (from the *x*-intercept of −0.4700 in Fig. 1C) in the presence of 1 µM lapatinib and *K*_M_ = 0.72 mM (from the *x*-intercept of −1.393 in Fig. 1C) in the absence of lapatinib. So, 1 µM lapatinib increased *K*_M_ of human mPGES-1 associated with GSH from 0.72 mM to 2.1 mM. As seen in Fig. 1C, the *y*-intercept = 0.15 ± 0.02 in the absence of lapatinib and the *y*-intercept = 0.18 ± 0.02 in the presence of 1 µM lapatinib; there was no significant difference in the *y*-intercept within the experimental errors. Hence, we can conclude that 1 µM lapatinib did not significantly change the V_max_, but significantly increased the *K*_M_ with respect to GSH, which is the well-known feature of the competitive inhibition. So, the experimental data depicted in Fig. 1C support that lapatinib indeed competes with co-factor GSH, which is consistent with the computationally modeled binding mode in which lapatinib occupies the GSH-binding site.

### *In vivo* anti-inflammatory activity of lapatinib in mice

**Figure 2:**
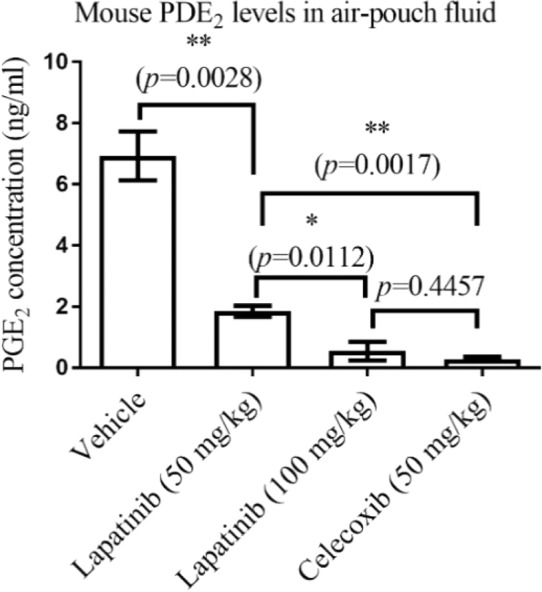
Data from *in vivo* assays using the mouse air-pouch model (n = 5 for each group) with lapatinib or celecoxib given PO. The air-pouch fluid collected from mice were analyzed by ELISA assay for the PGE_2_ concentrations. GraphPad Prism 7 software (GraphPad Software, La Jolla, CA) was used to perform the one-way analysis of variance (ANOVA) with *post hoc* tests.

To examine the anti-inflammatory potential of lapatinib, we determined the *in vivo* effectiveness of lapatinib using the most popularly used mouse air-pouch model of inflammation in comparison with celecoxib (a positive control). According to the *in vivo* data depicted in Fig. 2, lapatinib significantly and dose-dependently decreased the air-pouch PGE_2_ level in carrageenan-treated mice. In comparison with the positive control (celecoxib) at a dose of 50 mg/kg (PO), lapatinib at 50 mg/kg (PO) is less effective, as expected. Interestingly, there was no significant difference in the *in vivo* potency between 100 mg/kg lapatinib (PO) and 50 mg/kg celecoxib (PO), as shown in Fig. 2.

One can estimate the human equivalent dose (HED) associated with the highly effective mouse dose of 100 mg/kg by using the commonly used practice guide^37^ for dose conversion between different species:

1 $${\rm{HED}}=({{\rm{D}}}_{{\rm{animal}}}/{{\rm{K}}}_{{\rm{animal}}})={{\rm{D}}}_{{\rm{mouse}}}/12.3$$

in which K_animal_ is the correction factor (which is 12.3 for mouse) when the compound is equally potent against the human and animal target proteins. Further in consideration of the species difference in the *in vitro* potency, Eq. (1) can be extended in this study as the following:

2 $${\rm{HED}}=({{\rm{D}}}_{{\rm{mouse}}}/12.3)\times {{\rm{IC}}}_{50}({\rm{human}}\,{\rm{mPGES}}-1)/{{\rm{IC}}}_{50}({\rm{mouse}}\,{\rm{mPGES}}-1)$$

According to Eq. (2), one can estimate the HED associated with the mouse dose of 100 mg/kg: HED = (100/12.3) × (0.8/12) = ~0.54 mg/kg (or about 35 mg per dose for human with an average body weight of 65 kg). The *in vivo* data and the effective HED estimate suggest that lapatinib may serve as an effective anti-inflammatory drug to treat inflammation-related diseases for humans. It should be noted that this is merely an estimation based on the commonly used practice. The actual minimum effective dose for human can only be determined through actual clinical trials for further drug repurposing studies in the future.

### Insights from clinical data mining

As an FDA-approved drug, lapatinib was tested in many clinical trials. In clinical data mining, we would like to know whether lapatinib can reach the effective *in vivo* concentration in human body comparable to the IC_50_ value (~800 nM). To address this question, we collected the pharmacokinetic (PK) data, particularly the maximum drug concentration (C_max_) values, of lapatinib from five different clinical trials and estimated the corresponding C_max_ values in different tissues/organs. According to the data collected in Table 2, the plasma C_max_ of lapatinib in its usual dosage forms should be well above its IC_50_ against human mPGES-1. In addition, the reported ratios of C_max_ values in various organs of rat to C_max_ in rat plasma, along with the C_max_ values in human plasma, were used to predict the C_max_ values in the corresponding organs of human corresponding to the C_max_ values in human plasma. The predicted C_max_ values in various human organs are also listed in Table 2. As seen in Table 2, lapatinib may have much higher concentrations in various human organs compared to the corresponding concentration in human plasma. In particular, C_max_ is predicted to be as high as ~2 µM (> IC_50_) in lung and ~1.3 µM (>IC_50_) in kidney at a dose of only 50 mg. Notably, kidney is rich of mPGES-1^32^. All of these PK data suggest that lapatinib can serve as an effective anti-inflammatory drug to treat inflammation and pain as well as many other inflammation-related diseases.

**Table 2:** C_max_ (µM) of lapatinib in human plasma from clinical pharmacokinetic data and the predicted C_max_ (µM) in various human organs associated with various doses.

Dose	C_max_ (µM) in plasma	Predicted C_max_ (µM) in various organs^*e*^					
		Brain	Heart	Lung	Kidney	Intestine	Liver
10 mg	0.019^*a*^	0.002	0.041	0.311	0.201	0.100	0.002
25 mg	0.040^*a*^	0.004	0.085	0.649	0.420	0.210	0.005
50 mg	0.124^*a*^	0.012	0.267	2.037	1.319	0.658	0.015
100 mg	0.213^*a*^	0.021	0.458	3.500	2.266	1.131	0.026
175 mg	0.380^*a*^	0.038	0.817	6.243	4.043	2.018	0.046
250 mg	0.546^*a*^	0.055	1.174	8.971	5.809	2.899	0.066
500 mg	1.756^*b*^	0.176	3.775	28.851	18.684	9.324	0.211
650 mg	2.238^*b*^	0.224	4.812	36.770	23.812	11.884	0.269
900 mg	2.926^*b*^	0.293	6.291	48.074	31.133	15.537	0.351
1000 mg	3.184^*b*^	0.318	6.846	52.313	33.878	16.907	0.382
1250 mg	6.060^*c*^	0.606	13.029	99.566	64.478	32.179	0.727
1500 mg	8.598^*d*^	0.860	18.486	141.265	91.483	45.655	1.032

^a^Data in the row come from clinical trial by Bence *et al*.^38^. GW572016 ditosylate monohydrate formulated in oral suspension; ^*b*^Data in the row come from clinical trial EGF10004, Lapatinib (GlaxoSmithKline, Research Triangle Park, NC) was supplied as 100-mg and 250-mg tablets for daily oral administration; ^*c*^Data in this row come from clinical trial NCT00477464. Lapatinib was orally administered at 1250 mg once daily; ^*d*^Data in the row come from clinical trial NCT00486954 and NCT01138046, 36 data points from 6 subgroups were combined for the average C_max_. Lapatinib was in 6 pills at 250 mg each once oral daily; ^*e*^All of the predicted C_max_ values are calculated based on the ratios of the drug concentrations in various organs to that in plasma in rats reported in literature^56^.

## Discussion

As well known, mPGES-1 is very promising, but extremely difficult, target for practical drug development. A major difficulty exists in the difference in the PGH_2_-binding site of the enzyme between different species. For this reason, majority of the previously reported human mPGES-1 inhibitors are inactive against mouse or rat mPGES-1 such that one cannot use the well-established mouse/rat models of inflammation, pain, and other diseases for preclinical studies. Hence, it is interesting to explore novel inhibitors that can bind to the GSH-binding site which is more conserved for different species. This is the first report of an mPGES-1 inhibitor competitive with GSH. The competitive inhibition has been supported by the *in vitro* experiment. Further effort in this topic will be needed to obtain a co-crystal structure of mPGES-1 with lapatinib.

Our structure-based virtual screening of FDA-approved drugs, followed by *in vitro* and *in vivo* activity assays, has demonstrated that multiple FDA-approved drugs, including lapatinib, can significantly inhibit mPGES-1. Thus, these FDA-approved drugs could also have anti-inflammatory and analgesic effects, depending on their actual pharmacokinetic profiles associated with the actual dosage forms. Further clinical data mining revealed that lapatinib has a favorable pharmacokinetic profile to ensure that the lapatinib concentrations in various tissues can reach the levels that are significantly higher than the IC_50_ value after orally taking a lower dose of lapatinib. So, lapatinib may serve as a highly desired anti-inflammatory drug targeting mPGES-1 to treat inflammation and pain as well as a variety of other inflammation-related diseases. For example, the recently identified COVID-19 causes serious lung inflammation. It would be interesting to test whether lapatinib can be used to effectively suppress the inflammation caused by COVID-19 while an antiviral drug (such as remdesivir or chloroquine or hydroxylchloroquine or any other antiviral drug to be approved by the FDA) is used to kill the virus in the body.

It has been known that lapatinib has its anti-cancer activity through inhibition of epidermal growth factor receptor (EGFR) and human epidermal growth factor receptor 2 (HER2). So far, lapatinib has been used only for its anti-cancer activity in clinical practice. Hence, further clinical trials will be required before FDA approves repurposing lapatinib as an anti-inflammatory drug to treat inflammation and pain as well as a variety of inflammation-related diseases. Notably, reported phase I clinical trials in non-cancer patients^38^ indicated that lapatinib did not cause any serious adverse effects or significantly increase other common adverse effects, suggesting that lapatinib has the desirable safety as a novel anti-inflammatory drug for both cancer and non-cancer patients. In another Phase I dose-escalation study^39^, the highest dose of lapatinib used was as high as 7000 mg per day in twice-daily dosing with no dose-limiting toxicity (DLT) found, further demonstrating the safety of lapatinib. On the other hand, it is possible that lapatinib has side-effects that are not relevant in patients with cancer, but that can be important in patients with inflammatory diseases. The risk/benefit of drugs might be dependent on the clinical condition that is taken into consideration. So, it would be interesting to conduct drug repurposing clinical trials on lapatinib for its analgesic effects, because only clinical trials can determine its efficacy, as well as the safety, as an anti-inflammatory drug.

Concerning whether the mPGES-1 inhibition will indirectly increase the production of other PGs while decreasing the PGE_2_ production, there have been a variety of mPGES-1 knockout studies in mice. The genetic inactivation of mPGES-1 has been shown to result in the shunting of the PGH_2_ substrate to other terminal PG synthases, thereby increasing the synthesis of other PGs. Different reports showed that in explants of peritoneal macrophages isolated from mPGES-1-deficient mice that the reduction in PGE_2_ synthesis was accompanied by an increase in PGI_2_ and TXA_2_ stable metabolites^40^, increased PGI_2_ synthesis without increased TXA_2_ synthesis^41^, or no change in PGI_2_ and TXA_2_ synthesis^42^. Although PGD_2_ and PGF_2α_ production from peritoneal macrophages have also been shown to be altered following mPGES-1 deficiency, changes in these products have been shown to be less significant than the effects on PGI_2_ and TXA_2_^40^. Although a variety of studies using *in vitro* cell culture or cell explant culture systems have shown the potential for the shunting of PGH_2_ substrate following mPGES-1 inactivation, the causative effects of substrate shunting *in vivo* is less well defined. An adverse effect on cardiovascular function resulting from increases in thrombogenic TXA_2_ production or decreases in cardioprotective PGI_2_ is one area where changes in the PG synthetic profile following mPGES-1 inactivation has been of particular concern. However, an *in vivo* study examining cardiovascular function showed no increase in TXA_2_ synthesis, thrombosis, or blood pressure in mPGES-1-deficient mice, and an increase in cardioprotective PGI_2_ production, thereby suggesting the potential for mPGES-1 inhibitors to be effective anti-inflammatory agents without adverse cardiovascular effects^7^. There was also concern that reduced synthesis of PGE_2_ due to inactivation of mPGES-1 may also produce adverse effects in tissues where PGE_2_ is responsible for maintaining physiological function. Although the genetic deficiency of mPGES-1 was reported to decrease gastric PGE_2_ levels by approximately 80%^42^, the deficiency of mPGES-1 is not sufficient to induce gastric lesions. Additionally, our previous study in wild-type mice using a recently designed highly selective mPGES-1 inhibitor (4b)^32^ suggested that mPGES-1 should be a much safer target than COX-2, because 4b at a high dose (up to 5,000 mg/kg) administered (PO) did not cause any toxicity in mice while 50 mg/kg celecoxib (a selective COX-2 inhibitor) administered PO had significant toxicity for stomach and other issues of mice.

Further, the scaffold of the lapatinib structure may be used as a new starting point for future rational design of a lapatinib analog which can more potently inhibit mPGES-1 without inhibiting EGFR/HER2. Such a lapatinib analog may serve as an improved analgesic compared to lapatinib itself in terms of both the efficacy and side effects. Similarly, the scaffolds of other FDA-approved drugs identified as mPGES-1 inhibitors (with the IC_50_ values listed in Table 1) may also be used as new starting points for rational design of their analogs that can more potently and selectively inhibit mPGES-1.

In addition, the general DREAM-*in*-CDM approach may also be useful for repurposing FDA-approved drugs for other new therapeutic indications associated with new targets.

## Materials and Methods

### *In silico* simulation

The virtual screening of the FDA-approved drug from the Enzo Compound Library (http://www.enzolifesciences.com/BML-2843/screen-well-fda-approved-drug-library-v2/) in this study was based on the use of the recently reported open-conformation^35^ of human mPGES-1 and a revised version (with a unique feature) of our previously described computational protocol^33^. Prior to the virtual screening, the 3D structures of the FDA-approved drugs were generated by using OpenBabel^43^. Starting from the prepared structures, the computational protocol includes the use of AutoDock Vina 1.1.2 software^44^ for rigid molecular docking. A 15 Å × 15 Å × 15 Å docking box containing the original GSH-binding site of mPGES-1 was chosen as the targeted binding site and all other parameters were set to default. Only the top-ranked pose of the ligand was further evaluated using the AMBER16 software^45^. In all the subsequent simulations using the AMBER16, only ligand atoms and hydrogen atoms of the protein within 4 Å of the ligand were set flexible. For all the binding complexes, a four-step procedure, including 4,000 steps of energy minimization (1,000 steps with the steepest descend method and then 3,000 steps with the conjugate gradient method), 20 ps of MD simulation (temperature set to 300 K, time step was set to 2 fs, bonds involving hydrogen are constrained using SHAKE algorithm^46^ 6,000 steps of energy-minimization (2,000 steps with the steepest descend method and then 4,000 steps with the conjugate gradient method), and the molecular mechanics-Poisson-Boltzmann surface area (MM-PBSA) calculations^47^ (mbondi2 radii was used, parameter igb was set to 5, ionic strength was set to 0.1 mM, and external dielectric constant was set to 78.5.) for estimating the binding free energies. This multiple-step computational procedure is similar to the known binding estimation after refinement (BEAR)^48,49^ protocol. However, for a major difference between our current computational protocol and the BEAR protocol^48,49^, the free energy difference between the lowest-energy conformation of the free ligand and the conformation of the ligand in the mPGES-1-ligand binding complex was also calculated and added to the total binding free energy. Technically, the MM-PBSA binding free energy (ΔG_bind_) was evaluated as

3 $${{\rm{DG}}}_{{\rm{bind}}}={\rm{DG}}\,({\rm{complex}})-{\rm{DG}}\,({\rm{free}}\,{\rm{ligand}})-{\rm{DG}}\,({\rm{protein}})$$

In Eq. (3), as usual, ΔG (complex) and ΔG (protein) are the free energies of the protein-ligand complex and the free protein. However, ΔG (free ligand) is the free energy of the ligand in its free ligand conformation, instead of the ligand conformation existing in the protein-ligand complex. In this way, the calculated binding free energies are expected to be more reasonable, because they appropriately account for the conformational free energy change from the free-ligand conformation to the conformation in the protein-ligand binding complex. The compounds were then ranked by the estimated binding free energies and screened by the PAINS filter. According to the calculated binding free energies, the top-50 compounds were manually checked to remove the ones with clearly unreasonable ligand conformations, leading to selection of top-15 compounds.

In addition, the top-15 compounds were tested further for their binding with mPGES-1 in the high-resolution crystal structure (4AL0 with a high resolution at 1.2 Å) by using the same computational protocol (molecular docking, energy minimizations, MD simulations, and binding free energy calculations). Prior to the molecular docking, co-factor glutathione (GSH) in the crystal structure was removed from the binding site.

Molecular docking was also carried out for possible binding of lapatinib with COX-2 by using a reported X-ray crystal structure (PDB 5JW1)^50^.

### *In vitro* activity assays

The protocol for the protein preparation and *in vitro* activity assays were the same as described previously^51–54^. The enzyme activity assays were performed on ice in 1.5 ml microfuge tubes by using the expressed human or mouse mPGES-1. The reaction mixture contained: 0.2 M Na_2_HPO_4_/NaH_2_PO_4_, pH 7.2, 10 µL; 0.1 M GSH, 2.5 µL; diluted microsomal enzyme (80 µg/mL), 1 µL; PGH_2_ (0.31 mM in DMF), 5 µL; 1 µL inhibitor; and H_2_O in a final reaction volume of 100 µL. PGH_2_ was stored in dry ice and used to initiate the reaction. Compounds were incubated with the enzyme for 15 min at room temperature before the addition of cold PGH_2_ (1 µM final) to initiate the enzyme reaction. After 30 s, 10 µL of SnCl_2_ (40 mg/mL) in ethanol was added to stop the reaction. The non-enzymatic conversion of PGH_2_ to PGE_2_ was performed in the same buffer devoid of enzyme. The reaction mixture was placed on ice until PGE_2_ production was determined by the PGE_2_ enzyme immunoassay as described earlier. IC_50_ values of the inhibitors were calculated by using the GraphPad Prism 7.

The inhibitory activity of lapatinib (the most active mPGES-1 inhibitor identified in this study) against COX isoenzymes was determined by using the COX (ovine/human) inhibitor screening assay kit purchased from Cayman Chemical Company (Ann Arbor, MI). We used a mixture of equal amounts of purified COX-1 and COX-2 proteins and followed the protocol recommended by the vendor.

### *In vivo* activity tests

For *in vivo* tests, additional lapatinib sample (10 g at a purity of >99% determined by HPLC) was ordered from the LC laboratories (Woburn, MA). All the animal experiments were conducted in our animal laboratories in the University of Kentucky’s Division of Laboratory Animal Resources (DLAR) facility (PHS assurance number A3336-01; USDA number 61-R-0002; AAALAC, Intl. Unit # 13) following the guidelines and regulations of the AAALAC and National Institutes of Health (NIH). The animal procedure used in this project was approved by the University of Kentucky’s Institutional Animal Care and Use Committee (IACUC). The air-pouch model of inflammation^23,55^ is widely used for determining the *in vivo* effectiveness of inhibitors of prostaglandin synthesis. Air pouches were produced by duplicate injections of 3 mL of sterile air under the skin on the back of mice. After the formation of the air-pouch, a single injection of the inflammatory agent carrageenan into the pouch resulted in the recruitment of inflammatory cells and the production of a fluid exudate containing significant levels of PGE_2_ (an inflammatory marker) produced primarily by activities of COX-2 and mPGES-1. Then, the mice were treated PO (oral gavage) with a single dose of lapatinib, celecoxib, or vehicle for 24 hours prior to collection of air-pouch fluid samples. The air-pouch fluid samples were analyzed for PGE_2_ by the same ELISA method used in the *in vitro* enzyme activity assay mentioned above.

### Statistical analysis

GraphPad Prism 7 software (GraphPad Software, La Jolla, CA) was used to perform the one-way analysis of variance (ANOVA) with *post hoc* testing, allowing us to examine the significance of the difference in the *in vivo* activity data between each pair of dose conditions. *p* < 0.05 was considered statistically significant.

## Supplementary Information

Supplementary Information.
