# Supplementary material for: DREAM-in-CDM Approach and Identification of a New Generation of Anti-inflammatory Drugs Targeting mPGES-1
Source: Sci Rep. 2020 Jun 23;10:10187. doi: 10.1038/s41598-020-67283-0 (PMC7311425; doi:10.1038/s41598-020-67283-0)
Supplement: Supplementary file 1 — Supplementary Information. [file 41598_2020_67283_MOESM1_ESM.pdf]

Supporting Information

DREAM-*in*-CDM Approach and Identification of a New Generation of Anti-inflammatory Drugs Targeting mPGES-1

Shuo Zhou,[1,2,#] Ziyuan Zhou,[1,2,#] Kai Ding,[1,2,#] Yaxia Yuan,[1,2] Charles Loftin,[2] Fang Zheng,[1,2,*] and Chang-Guo Zhan[1,2,*]

[1]Molecular Modeling and Biopharmaceutical Center, College of Pharmacy, University of Kentucky, 789 South Limestone Street, Lexington, KY 40536. [2]Department of Pharmaceutical Sciences, College of Pharmacy, University of Kentucky, 789 South Limestone Street, Lexington, KY 40536

Molecular structures of the FDA-approved drugs listed in Table 1

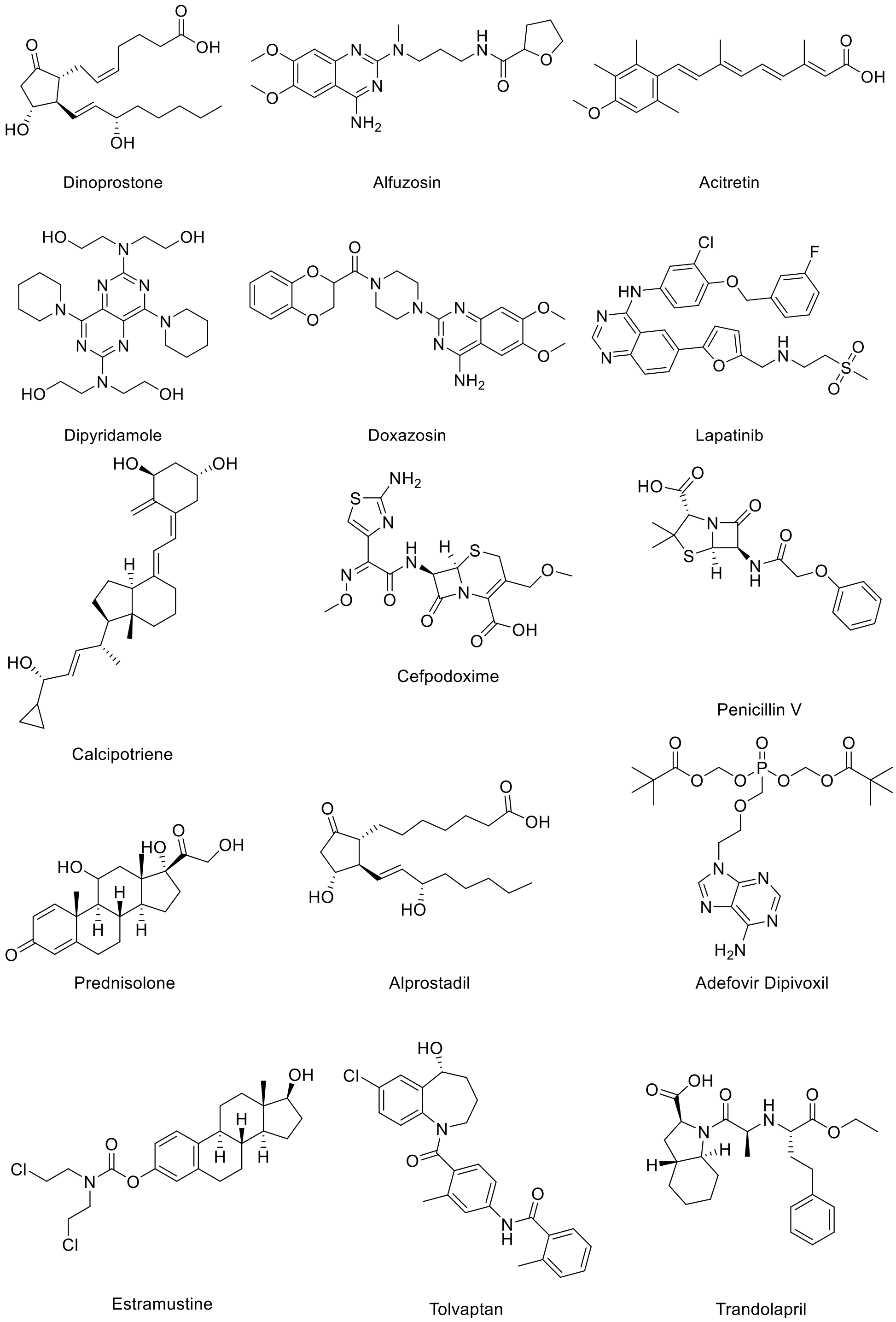

Figure S1. Molecular structures of the FDA-approved drugs listed in **Table 1**.
